# Neighborhood satisfaction, functional limitations, and self-efficacy influences on physical activity in older women

**DOI:** 10.1186/1479-5868-5-13

**Published:** 2008-02-27

**Authors:** Katherine S Morris, Edward McAuley, Robert W Motl

**Affiliations:** 1 University of Illinois at Urbana-Champaign, Urbana, IL, USA

## Abstract

**Background:**

Perceptions of one's environment and functional status have been linked to physical activity in older adults. However, little is known about these associations over time, and even less about the possible mediators of this relationship. We examined the roles played by neighborhood satisfaction, functional limitations, self-efficacy, and physical activity in a sample of older women over a 6-month period.

**Methods:**

Participants (*N *= 137, *M *age = 69.6 years) completed measures of neighborhood satisfaction, functional limitations, self-efficacy, and physical activity at baseline and again 6 months later.

**Results:**

Analyses indicated that changes in neighborhood satisfaction and functional limitations had direct effects on residual changes in self-efficacy, and changes in self-efficacy were associated with changes in physical activity at 6 months.

**Conclusion:**

Our findings support a social cognitive model of physical activity in which neighborhood satisfaction and functional status effects on physical activity are in part mediated by intermediate individual outcomes such as self-efficacy. Additionally, these findings lend support to the position that individual perceptions of both the environment and functional status can have prospective effects on self-efficacy cognitions and ultimately, physical activity behavior.

## Background

Older adults represent the fastest growing segment of the U.S. population, creating an increased prevalence of individuals at risk for, and living with, chronic disease and functional disability. Consequently, understanding those factors that promote physical, emotional, and psychological health among older adults is of importance. The beneficial effects of a physically active lifestyle on a variety of physical and psychological outcomes are well-established [1,2]. However, in spite of recent efforts to increase physical activity among older adults, relatively few older adults engage in leisure-time physical activity of sufficient frequency, duration, and/or intensity to elicit health benefits [3]. This trend toward sedentary behavior is even more marked among older adults with functional limitations [3].

Previous studies have examined the relationships between physical activity behavior and various individual, psychosocial, and environmental factors [4-8]. However, few studies have examined these relationships simultaneously and within a theoretical framework. Social Cognitive Theory [9] provides an excellent framework for examining these relationships positing that individual behavior is influenced by both personal and environmental factors. One important person factor is functional limitation. With advancing age the likelihood of developing functional limitations and disability increases [10,11]. Functional limitations are typically exhibited as perceived difficulty with walking, carrying, and lifting. Previous research has established an association between functional limitations and physical activity, such that individuals reporting more limitations report being less active [12,13].

Self-efficacy has been identified as another important personal factor influencing a myriad of health behaviors including physical activity [14] and reflects beliefs in one's ability to successfully carry out a course of action [15]. Importantly, there is evidence to suggest that efficacy expectations may provide an intermediary pathway between functional limitations and physical activity [14,15]. Thus, functional limitations influence physical activity indirectly, operating through self-efficacy.

Recently, there has been increased attention paid to the role of objective and perceptual components of the environment in influencing physical activity [16,17]. Individual perceptions of the built environment are believed to both foster and inhibit a physically active lifestyle [15,18,19]. In addition to perceptions of sidewalk conditions and proximity to facilities [17,20], perceived neighborhood satisfaction has commonly been assessed along with physical activity behavior [21,22]. Social cognitive theory would posit that perceptions of the environment act as a source of self-efficacy information. That is the presence of a facilitative or restrictive environment influences efficacy expectations which, in turn, drive behavior [23].

Prodaniuk and colleagues [24] examined this mediational relationship in their study of the associations between outcome expectations, self-efficacy, and perceptions of the workplace environment using bivariate and multiple regression analyses of cross-sectional data. This study reported physical activity and self-efficacy to be weakly correlated with environmental perceptions. Subsequent mediational analyses in which self-efficacy was controlled, demonstrated a decrease in the magnitude of the environmental effect on physical activity from β = .23 to β = .16. The amount of variance in physical activity accounted for by the perceived environment also decreased as a result of including self-efficacy as a mediating variable in the model from 4% to 2%.

Further evidence of a mediatonal relationship is reported in a recent study conducted by Motl and colleagues [25] using cross-sectional and longitudinal data in a sample of adolescent girls. A significant cross-sectional relationship between equipment accessibility and self-efficacy was demonstrated, as was a significant relationship between self-efficacy and physical activity. However, there was no direct relationship between neighborhood safety or equipment accessibility and physical activity, suggesting a mediating effect of self-efficacy. This pattern of relationships was not strongly supported in the longitudinal analysis, as self-efficacy only weakly mediated the effects of equipment accessibility (*β *= .03) and neighborhood safety (*β *= .02) on physical activity.

A social cognitive perspective would view both person and perceived environmental factors as playing a role in shaping physical activity behavior. Such influence would be expected to operate through perceptions of personal efficacy. That is, individuals with fewer functional limitations and who are more satisfied with their neighborhood are likely to be more efficacious. In turn, more efficacious individuals are likely to be more active. Thus, the primary objective of this study was to prospectively examine the role of three perceptual variables: self-efficacy, functional limitations, and neighborhood satisfaction in determining changes in physical activity in a sample of older women. As specific aspects of the built environment (i.e., access to services, safety) are encompassed within judgments of neighborhood satisfaction [26], we chose to focus on the role of neighborhood satisfaction in predicting physical activity behavior. Our use of neighborhood satisfaction is appropriate in light of our research question; examining the respective contributions of perceptual variables on physical activity behavior. That is, it is an individual's personal assessment of his or her environment–rather than the judgment of others demonstrated by environmental audits–which influences individual behavior in that environment.

Finally, this study is a response to previous calls for research examining both direct and indirect pathways between both perceived and objective aspects of the environment and physical activity behavior [16,27]. All hypothesized relationships were tested in a 6-month prospective observational design.

## Methods

### Participants

Older women (*N *= 137) were recruited from an on-going prospective study of older women's health via an announcement in the project newsletter which described this separate, mail-based study.

### Measures

#### Physical activity

Physical activity was assessed using the Physical Activity Scale for the Elderly [PASE; [28]], a self-report measure frequently used to assess physical activity in older adults [29,30]. This 10-item self-report questionnaire asks participants to record activity participation over the previous 7 days and has been widely used to assess physical activity in older adults. The PASE contains activities from several domains including lifestyle/leisure (e.g., walking outside of the home), household (e.g., light housework, outdoor gardening), and occupation (e.g., work done primarily while sitting). The total PASE score is computed by multiplying the amount of time spent in each activity (hours/week) or participation (yes/no) in an activity by the empirically derived item weights (syntax is on following page) and summing over all activities. Previous studies have reported PASE scores ranging from 0–312 in samples of older adults, with an average PASE score of 119 [30]. This scale has demonstrated reliability and validity among community-dwelling older adults [31] and older adults with osteoarthritis [29].

#### Functional limitations

The abbreviated version [32] of the *Advanced Lower Extremity Function *component of the Late-Life Function and Disability Instrument [LL-FDI; [33,34]] was used to report difficulty in performing various tasks (e.g., walking one mile without stopping to rest). Responses on this 5-item subscale are rated on a Likert scale ranging from 1 (cannot do) to 5 (none), with higher scores reflecting less difficulty in performing tasks. Items are summed to arrive at a total score for lower extremity function, resulting in a scale score ranging from 5–25. This subscale has demonstrated reliability among older adults [32,35]. Internal consistencies of this scale at Baseline (α = 0.88) and Month 6 (α = 0.86) were strong.

#### Perceptions of Neighborhood Satisfaction

The *Neighborhood Satisfaction *subscale of the Neighborhood Environment Walkability Scale [NEWS; [36]] was used as a composite measure to assess perceptions of environment quality. This measure is comprised of 17 items scored on a scale from 1 (strongly dissatisfied) to 5 (strongly satisfied). Example items include satisfaction with "how easy and pleasant it is to walk in your neighborhood," and "access to shopping in your neighborhood." The scores across all items were summed and divided by the number of items to arrive at a total scale score. Higher scores indicated greater levels of satisfaction with their neighborhood characteristics. The range of the total scale value is 1–5. Internal consistencies for this subscale at both time points (α > 0.86) were acceptable.

#### Self-efficacy

The 13-item *Barriers Self-Efficacy Scale *[BARSE; [37]] was used to assess individuals' beliefs in their capability to exercise three times per week for 40 minutes over the next three months in the presence of commonly cited barriers to participation. Example items include confidence to exercise 3 times per week for 3 months if "the weather was very bad" and "I had to exercise alone." Responses were recorded using a percentage scale ranging from 0% (not at all confident) to 100% (highly confident) in 10-point increments. The scores across all items were summed and divided by the number of items to arrive at a total scale score. The range of the total scale value was 0 to 100. Internal consistencies for this scale at Baseline (α = 0.96) and Month 6 (α = 0.96) were excellent.

### Procedures

All participants completed an Institutional Review Board-approved informed consent prior to completing study materials. All measures were distributed and collected at baseline and 6-months through the mail with the use of self-addressed, pre-paid envelopes.

### Data Analysis

We conducted a panel analysis using covariance modeling with full-information maximum-likelihood (FIML) estimation in Mplus 4.1 [38]. The panel analysis allowed for examinations of cross-sectional relationships with baseline data and longitudinal associations across 6 months. The examination of longitudinal associations is accomplished based on residual changes in model constructs over time by controlling for initial baseline relationships using stability paths (i.e., paths linking the same construct across time). Covariance modeling was chosen over other, more traditional [39] tests of mediating variable effects as we were very much interested in testing the *theoretical pattern *of relationships specified; not simply testing each pathway alternately. Recent simulation studies of 14 different methods of testing mediation demonstrate that incremental tests (i.e., completion of multiple regressions) had low power to detect small and medium effects [40]. Based upon these simulation studies, covariance modeling techniques within a structural equation model framework is the preferred method for assessing mediation effects as it allows one to test the plausibility of competing sequential models while retaining statistical power [40]. There was no loss to follow-up across the two time points of this study, providing us with a complete data set free of missing data.

#### Model specification

**Figure 1:**
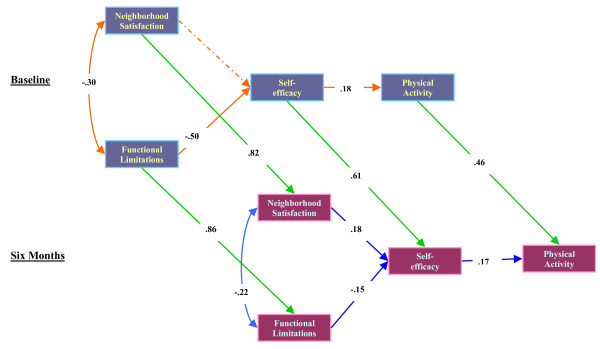
Panel model showing mediated effects of neighborhood satisfaction and functional limitations on physical activity at baseline (upper panel) and at 6 months (lower panel). *Note*: Relationships at 6 months reflect changes over time. Solid lines represent significant parameter estimates, and dashed lines represent nonsignificant parameter estimates.

Figure 1 shows the panel model tested and includes: (a) paths from neighborhood satisfaction and functional limitations to self-efficacy at both baseline and 6 months; (b) a path from self-efficacy to physical activity at both baseline and 6 months; (c) and paths between the same variables measured across time (i.e., stability coefficients). Therefore, baseline data involves cross-sectional associations and 6-month data involves the study of changes among constructs. The model further included correlations between neighborhood satisfaction and functional limitations at baseline and 6-month follow-up.

#### Model fit

Model-data fit was assessed using the chi-square statistic, root mean square error of approximation (RMSEA), and comparative fit index (CFI). The chi-square statistic assessed absolute fit of the model to the data [41]. The RMSEA represents closeness of fit, and values approximating .06 or less reflect a good fitting model [42,43]. The CFI tested the proportionate improvement in fit by comparing the hypothesized model with the independence model [43]. Values approximating 0.95 or greater indicate a good fitting model [43].

## Results

The mean age of the sample was 69.6 years, 84% of the participants were White, a majority of the participants were married, 46% had received a university degree, and 46% reported an income greater than $40,000. Although community-dwelling, many of these women reported medical diagnoses of hypertension (36.6%), hyperlipidemia (27.6%), and functional impairment of the musculoskeletal system (85.1%).

Table 1 presents the mean and standard deviation values for all variables and Table 2 provides a correlation matrix of all bivariate associations among study variables. As can be seen from the mean scale scores presented in Table 1, this sample of older women reported substantial limitations in advanced lower extremity function as evidenced in a response of "cannot do" or "quite a lot of difficulty" on 4 of the 5 scale items by >70% of the participants. Conversely, these women were quite satisfied with the walkability of their neighborhoods, were moderately confident in their ability to overcome barriers, and engaged in low/moderate amounts of leisure-time physical activity. The hypothesized panel model provided a good fit to the data, χ^2^(14) = 17.56, *p *= .23, CFI = .98, RMSEA = .04. With the baseline assessment, standardized parameter estimates indicated that functional limitations had a direct effect on self-efficacy (γ = -.50). In turn, self-efficacy had a direct effect on physical activity (β = .26). Individuals with fewer limitations reported higher levels of self-efficacy and in turn reported being more physically active. The relationship between neighborhood satisfaction and self-efficacy was non-significant (γ = .11). The indirect effects of neighborhood satisfaction (γβ = .02) and functional limitations (γβ = -.09) on physical activity were relatively small.

**Table 1:** Descriptive Statistics for the Measures of Neighborhood Satisfaction, Functional Limitations, Self-Efficacy, and Physical Activity Across the Two Timepoints

	*Baseline Assessment*		*6-Month Assessment*	
	*M*	*SD*	*M*	*SD*
Neighborhood Satisfaction	3.8	0.6	3.8	0.7
Functional Limitations	11.2	4.8	11.4	4.7
Self-efficacy	64.3	25.2	58.5	26.5
Physical Activity Scale for the Elderly	136.5	64.0	147.8	62.9

**Table 2:** Correlation Coefficients Among the Measures of Neighborhood Satisfaction, Functional Limitations, Self-Efficacy, and Physical Activity Across the Two Timepoints

	1	2	3	4	5	6	7	8
1. Neighborhood Satisfaction, baseline assessment	--							
2. Functional limitations, baseline assessment	-.300	--						
3. Self-efficacy, baseline assessment	.260	-.530	--					
4. PASE, baseline assessment	.148	-.240	.261	--				
5. Neighborhood Satisfaction, 6-month assessment	.825	-.247	.203	.119	--			
6. Functional limitations, 6-month assessment	-.297	.864	-.501	-.190	-.221	--		
7. Self-efficacy, 6-month assessment	.319	-.454	.721	.245	.337	-.496	--	
8. PASE, 6-month assessment	.141	-.272	.321	.526	.139	-.341	.360	--

*Note*. Correlations were computed using the saturated model and full-information maximum- likelihood estimation in Mplus 4.1.

PASE = Physical Activity Survey for the Elderly.

Relative to relationships among changes over time, changes in neighborhood satisfaction and functional limitations had significant direct effects on residual changes in self-efficacy (β = .18, β = -.15, respectively), and changes in self-efficacy were significantly associated with changes in physical activity (β = .25) at Month 6. Thus, increases in neighborhood satisfaction and perceived function were associated with increased self-efficacy, and increases in perceived function and self-efficacy were, in turn, associated with being more physically active over time. In addition, the stability coefficients were acceptable for neighborhood satisfaction (γ = .82), functional limitations (γ = .86), self-efficacy (β = .61), and physical activity (β = .47). Neighborhood satisfaction and functional limitations were significantly correlated at the baseline assessment (Φ = -.30), and at 6-months (ψ = -.22). Individuals with fewer functional limitations reported greater neighborhood satisfaction. The indirect effects of changes in neighborhood satisfaction (γβ = .03) and functional limitations (γβ = .03) on physical activity were small. In total, the model accounted for 7% of the variation in physical activity at baseline and 33% of the variation in change in physical activity at 6 months. This model is shown in Figure 1.

We conducted a second series of analyses to examine the utility of including direct paths between physical activity and neighborhood satisfaction and functional limitations. This model provided a slightly better fit for the data, χ^2^(10) = 10.75, *p *= .38, CFI = .99, RMSEA = .02 than the model which specified only indirect effects. However, the difference between the two models was not statistically significant χ^2^(4) = 6.81, *p *> .10. The only additional path that was significant was between changes in functional limitations and changes in physical activity (γ = -.17). This in turn, attenuated the effect of changes in self-efficacy on changes in physical activity (β = .17). In total, this model accounted for 9% of the variation in physical activity at baseline and 35% of the variation in change in physical activity at 6 months.

## Discussion

The primary objective of this study was to prospectively examine the role of individual, psychosocial, and perceived environmental factors in determining changes in physical activity in a sample of older women. The present findings suggest that perceived neighborhood satisfaction and functional limitations are associated with changes in physical activity behavior. Importantly, these effects are, in part, mediated by self-efficacy. The physical activity behavior literature has largely focused on the effects of individual-level variables [44], with more recent efforts examining the role of the perceived and built environment. The tendency, however, is to examine these factors separately [18,24,25]. As Duncan et al. [45] note, the failure to develop and evaluate more comprehensive models of physical activity is likely to influence both the magnitude and the nature of these associations.

Our results are consistent with a social cognitive perspective, such that perceptions of neighborhood satisfaction and functional limitations operate as sources of efficacy information [15]. We found that older adults who reported greater neighborhood satisfaction and fewer lower extremity limitations also had higher levels of efficacy to overcome barriers to exercise over time. Additionally, neighborhood satisfaction was inversely associated with functional limitations, suggesting that older adults who experience less difficulty with mobility and balance are more likely than those with greater limitations to report greater levels of neighborhood satisfaction. As hypothesized, self-efficacy was directly associated with physical activity. Although not hypothesized, the direct effect of functional limitations on physical activity behavior is not completely incompatible with a social cognitive perspective and lends support to findings from earlier studies [13].

The results of this study suggest that perceptions of neighborhood satisfaction do indeed influence physical activity behavior, albeit, through self-efficacy. Although this study is limited to the examination of perceptual variables, future investigations into the determinants and outcomes associated with neighborhood satisfaction are warranted. Specifically, investigations which objectively assess characteristics of the environment along with perceived neighborhood satisfaction are needed. Such research would inform intervention studies that target elements of the environment to improve health behaviors by identifying those aspects of the built environment which influence individual perceptions of neighborhood satisfaction, and ultimately, physical activity behavior.

Although our findings suggest that individual and psychosocial variables and neighborhood satisfaction may have roles to play in determining physical activity, several limitations should be considered when interpreting these results. First, these relationships were examined using a relatively small sample and only among older women. Second, this study relied on self-report measures of both physical activity and neighborhood satisfaction. However, it is important to note that this study was designed to test a social cognitive model of physical activity behavior, in which a mediating effect of self-efficacy was hypothesized. Furthermore, in their examination of environment characteristics and physical activity among older adults, Giles-Corti and Donovan [16] report that it was not the objective characteristics of the built environment, but individual perceptions, that were associated with physical activity.

The results of this study are also limited by the relative stability evidenced in activity levels over the course of the 6 months. However, such stability is in part, to be expected, as this was a prospective study, with no intervention made to alter behavioral patterns. Additionally, the relative stability observed in mean activity levels over the course of the six months implies little/no individual-level change. However, 25% of the study sample demonstrated either declines or increases in physical activity levels at or above 1 standard deviation. Similar rates of change were observed for other model variables. Studies of longer duration, in which greater individual variability in functional limitations and activity levels can be expected, are needed. Finally, the predictor variables included in this model were fairly restrictive. Neighborhood satisfaction is just one of several environmental factors thought to be associated with physical activity [45]. Additionally, our use of neighborhood satisfaction as a 'marker' for the built environment needs to be considered. Neighborhood satisfaction is a composite measure encompassing perceptions of environmental attributes such as safety, lighting, and aesthetic conditions. As such, it is not clear from these analyses which aspects of the environment are most salient in influencing self-efficacy, and ultimately, physical activity behavior. Future studies in which aspects of the built and social environment are more clearly specified are warranted.

## Conclusion

In closing, our study examined the relationships between the perceived environment, individual variables, and physical activity within the context of a theoretical framework and identified self-efficacy as a potential mediating variable in this relationship. We further believe this to be an important starting point in attempts to more extensively examine the joint influence of environmental and individual factors on physical activity behavior. Our results offer preliminary evidence for the role of both individual- and community-level variables in changes in activity behavior. However, longitudinal research that includes a wider variety of environmental variables in concert with psychosocial variables will do much to inform future behavior change endeavors.

## Competing interests

The author(s) declare that they have no competing interests.

## Authors' contributions

EM developed the theoretical framework and design of the study presented here and performed the statistical analyses. KM contributed to the design of the present study, supervised all data collection, and performed statistical analyses. RM served as a consultant for the statistical analyses. All authors made substantial contributions to drafting this manuscript.
